# Supplementary material for: Pattern of progression and post-progression survival following transarterial embolisation: An analysis of the TACE-2 and TACTICS trials
Source: JHEP Rep. 2026 Feb 25;8(5):101791. doi: 10.1016/j.jhepr.2026.101791 (PMC13081179; doi:10.1016/j.jhepr.2026.101791)
Supplement: Multimedia component 1 [file mmc1.pdf]

Pattern of progression and post-progression survival following transarterial embolisation: An analysis of the TACE-2 and TACTICS trials

Jack Shi Jie Yuan, Memuna Rashid, Kazuomi Ueshima, Andre Lopes, Yuk Ting Ma, Paul J Ross, Daniel Palmer, Masatoshi Kudo, Tim Meyer

Table of contents

Table S1……………………………………………………………………………………2

Table S2……………………………………………………………………………………3

Table S3……………………………………………………………………………………4

Table S4……………………………………………………………………………………4

Fig. S1…………………………………………………………………………………......5

Table S1: Baseline characteristics among patients with disease progression by treatment group

Baseline characteristics	TACE + Sorafenib N=140	TACE Control N=145
**Sex**		
Male	112 (80%)	117 (81%)
Female	28 (20%)	28 (19%)
**Age (years)**		
Median(range)	70.0 (36.0 to 85.0)	71.0 (46.0 to 86.0)
**Bilirubin (µmol/l)**		
Median(range)	13.0 (3.0 to 36.0)	14.0 (4 to 50)
**Albumin (g/l)**		
Median(range)	40.0 (29.0 to 50.0)	39.0 (26.0 to 57.0)
**RECIST: Target Lesion 1 (cm)**		
Median (range)	4.0 (1.0 to 19.6)	3.5 (1.0 to 23.0)
**RECIST: Target Lesion 2 (cm)**		
Median (range)	1.9 (0 .8 to 7.5)	2.0 (0.5 to 10.8)
**AFP (kU/L)**		
Median (mean) (range)	15.9 (2364.0) (0.8 to 100199.0)	14.8 (1988.6) (1.3 to 100000.0)
**Time from randomisation to progression(months)**		
Median (range)	7.1 (1.0 to 69.7)	5.4 (0.8 to 46.0)
**ECOG PS**		
0	104 (74%)	108 (74%)
1	35 (25%)	37 (26%)
Not known	1 (1%)	0 (0%)
**Disease Focality nodules**		
1	34 (24%)	37 (26%)
2	36 (26%)	35 (24%)
3	28 (20%)	23 (16%)
>3	41 (29%)	47(32%)
Not known	1(1%)	3(2%)
**HAP score**		
HAP A	59 (42%)	50 (34%)
HAP B	49 (35%)	61 (42%)
HAP C	26 (19%)	27 (19%)
HAP D	6 (4%)	4 (3%)

Not available	0 (0%)	3 (2%)

Table S2. Descriptive analysis of post-progression overall survival stratified by time from randomisation to progression

Time from randomisation to progression	N	N deaths (%)	Median PPOS (in months)	12 months PPOS
<6 months	144	118 (82%)	17.3	62%
≥6 months to ≤12 months	78	50 (64%)	23.2	72%
>12 months	63	35 (56%)	19.3	59%

*PPOS: Post-progression overall survival

Table S3. Post-progression overall survival by time from randomisation to progression groups (Univariable vs. Multivariable Cox Regression)

**Time from randomisation to progression**	**Univariable Cox Model**		**Multivariable Cox Model***	
	**HR (95% CI)**	**P-value**	**HR (95% CI)**	**P-value**
<6 months	1.00 (ref)		1.00 (ref)	
≥6 months to ≤12 months	0.87 (0.62 to 1.21)	0.41	0.87 (0.62 to 1.21)	0.41
>12 months	0.86 (0.59 to 1.26)	0.45	0.95 (0.65 to 1.3940)	0.80
HAP score				
HAP A	1.00 (ref)		1.00 (ref)	
HAP B	1.3029 (0.95 to 1.80)	0.104	1.30 (0.94 to 1.79)	0.11
HAP C	2.09 (1.44 to 3.05)	P < 0.001	2.08 (1.42 to 3.03)	p < 0.001
HAP D	13.89 (5.69 to 33.87)	P < 0.001	14.00 (5.71 to 34.33)	p < 0.001

* Adjusted for HAP score

Table S4. Comparison of the multivariable analysis of the TACE-2 and TACTICS trial

**Post-Progression Overall Survival (Multivariable model)**	TACTICS		TACE-2	
	HR (95% CI	P-value	HR (95% CI)	P-value
**Pattern of progression**				
Target or non-target lesion	1.00 (ref)		1.00 (ref)	
New lesion in the liver	1.45 (0.97 to 2.17	0.072	0.86 (0.51 to 1.43)	0.56
New lesion outside the liver	9.29(3.14 to 27.52)	p <0.001	1.35 (0.74 to 2.45)	0.33
HAP score				
HAP A	1.00 (ref)		1.00 (ref)	
HAP B	1.25 (0.84 to 1.85)	0.276	1.40 (0.79 to 2.46)	0.25
HAP C	1.48 (0.84 to 2.60)	0.176	2.55(1.41 to 4.60)	0.002
HAP D	-	-	9.04 (3.42 to 23.88)	p < 0.001

Fig. S1 – Kaplan Meier plot for post-progression overall survival stratified by time from randomisation to progression

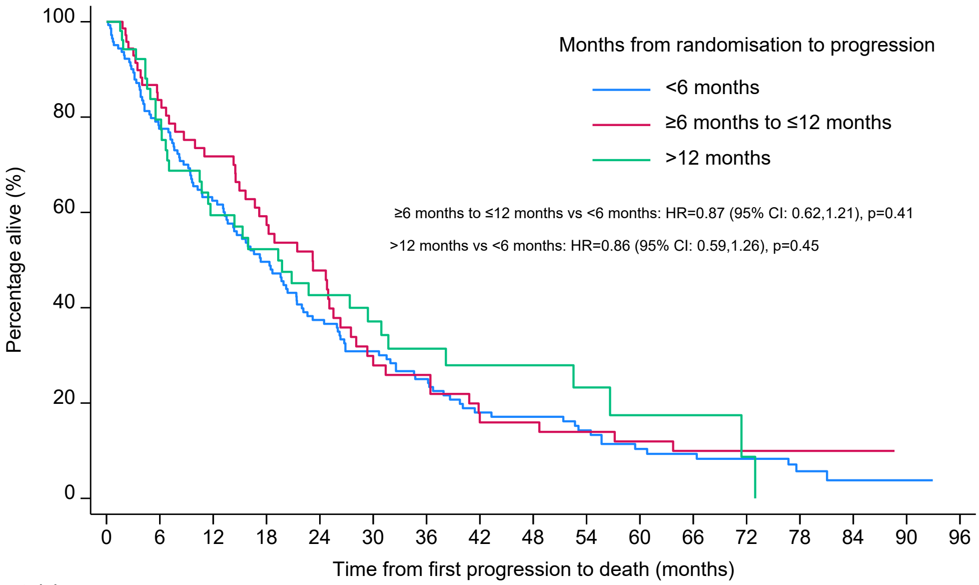

Number at risk																	
<6 months	144	104	80	62	46	37	30	20	18	15	10	9	7	4	2	1	0
≥6 months to ≤12 months	78	52	40	32	24	14	13	8	8	7	6	4	2	1	1	0	0
>12 months	63	37	25	22	16	13	10	8	7	4	2	2	1	0	0	0	0
