# Supplementary material for: Pattern of progression and post-progression survival following transarterial embolisation: An analysis of the TACE-2 and TACTICS trials
Source: JHEP Rep. 2026 Feb 25;8(5):101791. doi: 10.1016/j.jhepr.2026.101791 (PMC13081179; doi:10.1016/j.jhepr.2026.101791)
Supplement: Multimedia component 4 [file mmc4.pdf]

Research article

Pattern of progression and post-progression survival following transarterial embolisation: An analysis of the TACE-2 and TACTICS trials

Authors

Jack Shi Jie Yuan-Doré, Memuna Rashid, Kazuomi Ueshima, …, Daniel Palmer, Masatoshi Kudo, Tim Meyer

Correspondence

t.meyer@ucl.ac.uk (T. Meyer), m-kudo@med.kindai.ac.jp (M. Kudo).

Graphical abstract

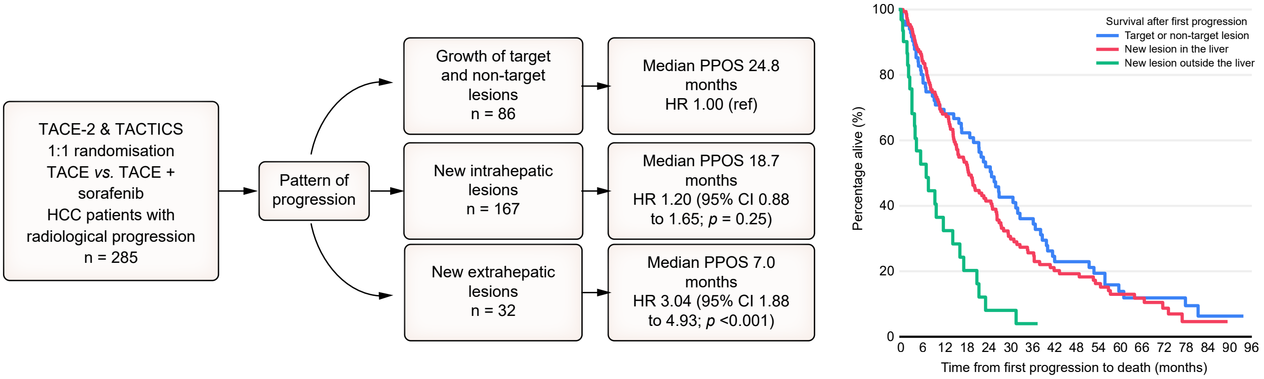

Highlights:

- Pattern of progression is an independent prognostic factor for overall survival in TACE-treated patients with HCC.
- TACE-treated patients who progress with extrahepatic progression have worse overall survival.
- Pattern of progression following TACE should be considered as a stratification factor in subsequent trials.

Impact and implications:

Pattern of progression has been validated as a significant independent prognostic factor for post-progression overall survival in patients with hepatocellular carcinoma (HCC) treated with systemic anticancer therapies and selective internal radiation therapy. This study is the first prospective multicentre analysis to demonstrate that pattern of progression is also a significant independent prognostic factor for post-progression overall survival in patients with HCC treated with transarterial chemoembolisation. Future trials of locoregional therapies for HCC should report pattern of progression and consider its use as a stratification factor in subsequent studies.

https://doi.org/10.1016/j.jhepr.2026.101791
© 2026 The Author(s). Published by Elsevier B.V. on behalf of European Association for the Study of the Liver (EASL). This is an open access article under the CC BY license (http://creativecommons.org/licenses/by/4.0/).

Pattern of progression and post-progression survival following transarterial embolisation: An analysis of the TACE-2 and TACTICS trials

Jack Shi Jie Yuan-Doré[1], Memuna Rashid[2], Kazuomi Ueshima[3], Andre Lopes[2], Yuk Ting Ma[4], Paul Ross[5], Daniel Palmer[6], Masatoshi Kudo[3,*], Tim Meyer[1,7,*]

JHEP Reports **2026**. vol. 8 | 1–8

Check for updates

Background & Aims: Previous studies have demonstrated that the pattern of progression (POP) following systemic therapy or transarterial radioembolisation is prognostic for post-progression overall survival (PPOS). POP has therefore been proposed as a stratification factor for subsequent clinical trials. However, its significance in TACE-treated populations has not been prospectively explored. We analysed the impact of POP on PPOS in patients treated in the TACE-2 and TACTICS trials.

Methods: TACE-2 and TACTICS are two prospective, multicentre, randomised trials comparing TACE plus sorafenib with TACE plus placebo, conducted in the UK and Japan, respectively. Patients with radiological progression in both trials were included in this analysis. POP was defined as: target or non-target lesion progression (TNTLP), new intrahepatic lesions (NIH), or new extrahepatic lesions (NEH). As extrahepatic disease was an exclusion criterion in both trials, only intrahepatic lesions qualified for the TNTLP group. PPOS was assessed using the Kaplan–Meier method, and comparisons were performed using the log-rank test.

Results: A total of 285 patients were included (86 with TNTLP, 167 with NIH, and 32 with NEH). Median PPOS was 24.8 months for TNTLP, 18.7 months for NIH, and 7.0 months for NEH. Compared with the TNTLP group, patients with NEH had significantly worse survival (adjusted hazard ratio 3.05, 95% CI 1.88–4.94; p <0.001), whereas survival in the NIH group was not significantly different (adjusted hazard ratio 1.21, 95% CI 0.88–1.65; p = 0.24). No survival differences were observed between placebo and sorafenib arms within each POP group.

Conclusion: Progression in the form of new extrahepatic disease following TACE occurred in only 11% of patients but was associated with poor overall survival, reinforcing the importance of extrahepatic disease as a stratification factor in clinical trials.

© 2026 The Author(s). Published by Elsevier B.V. on behalf of European Association for the Study of the Liver (EASL). This is an open access article under the CC BY license (http://creativecommons.org/licenses/by/4.0/).

Introduction

Transarterial embolic therapy is considered a standard of care for selected patients with liver-confined hepatocellular carcinoma, preserved liver function and good performance status[1–4] in whom it has been shown to improve survival compared with best supportive care.[5] The methodology for embolic therapy remains highly variable[6] and conventional TACE (cTACE), drug-eluting bead TACE (DEB-TACE) and bland transarterial embolisation (TAE) are all considered acceptable techniques, with no convincing evidence of superiority of one method over another.[7,8]

The prognosis for patients undergoing embolic therapy is determined by both tumour factors and liver function. Composite scores such as the hepatoma arterial embolisation prognostic (HAP) score[9] allow for prognostic stratification at baseline, while other models have included treatment response to provide prognostic estimates post-therapy.[10] What has been less well established is the prognostic impact of pattern of progression after embolic therapy. Whilst this has been explored in a limited number of small, single-centre retrospective analyses,[11,12] there have been no large, prospective multicentre studies.

TACE-2 and TACTICS were randomised controlled trials comparing TACE and sorafenib *vs.* TACE plus placebo or TACE alone.[13–15] Both studies failed to demonstrate an improvement in overall survival with the addition of sorafenib to TACE. Collectively, these studies recruited 469 patients from 53 centres in the UK and Japan. Here, we report a combined analysis evaluating the prognostic impact of pattern of progression on post-progression survival. Uniquely, we also assess the impact of systemic therapy with sorafenib on pattern of progression and post-progression overall survival (PPOS).

Patients and methods

Study design and patients

This is a *post hoc* analysis of prospective data acquired from TACE-2 and TACTICS trials. The aim of the study was to

* Corresponding authors. Addresses: Department of Medical Oncology, Royal Free Hospital, London, NW3 2QG, UK (T. Meyer), or Department of Gastroenterology and Hepatology, Kindai University Faculty of Medicine 377-2, Ohno-Higashi, Osaka-Sayama Osaka, Japan (M. Kudo).
E-mail addresses: t.meyer@ucl.ac.uk (T. Meyer), m-kudo@med.kindai.ac.jp (M. Kudo).
https://doi.org/10.1016/j.jhepr.2026.101791

examine the association between pattern of progression on PPOS in patients who had disease progression after receiving TACE treatment. The primary endpoint of this study was PPOS, defined as the time from progression to death. The study population included patients from the TACE-2 and TACTICS trials who had radiological disease progression according to the criteria specified in their respective protocols. All patients who had documented disease progression at the time of trial closure were included. The study population therefore included patients who had progression as best response but also those who had subsequently progressed after stable disease or response. Those patients that progressed after closure of the trial were not included since data was not collected beyond the date of trial closure. The pattern of progression was recorded on the trial case report form based on local radiological review. For TACE-2, there was also central radiological review to confirm the pattern of response. The case report form did not distinguish between macrovascular invasion and metastatic disease; therefore, both are included in the classification of new extrahepatic lesions (NEH).

TACE-2 (NCT01324076) and TACTICS (NCT01217034) were randomised studies whose design and patient eligibility have previously been reported.[13–15] In brief, both studies included patients meeting the standard criteria for TACE, namely those diagnosed with HCC based on AASLD criteria who were not candidates for surgical resection or transplant, without vascular invasion and extrahepatic metastases, ECOG performance status of 0 or 1, and well-preserved organ function. Of note, both studies included patients with Child-Pugh class A liver function, but the TACTICS trial also included patients with Child-Pugh class B7 liver function.

Both studies randomised patients in a 1:1 ratio of TACE plus sorafenib *vs.* TACE plus placebo (TACE-2) or TACE alone (TACTICS). In the TACE-2 trial, sorafenib was started at a dose of 400 mg twice-daily within 24 h of randomisation and TACE was performed 2-5 weeks post-randomisation. In TACTICS, sorafenib was started at 400 mg per day and TACE performed 2-3 weeks later with the option to increase to 800 mg per day at the discretion of the investigator. The TACE-only arm within TACTICS was not placebo-controlled. For the TACE treatments, the TACE-2 trial used DEB-TACE with drug-eluting beads loaded with 150 mg of doxorubicin, whereas the TACTICS trial employed cTACE, administering lipiodol with epirubicin or a miriplatin suspension, followed by Gelpart embolic agent.

In TACE-2, the primary endpoint was progression-free survival (PFS), with progression defined according to RECIST v1.1.[16] OS and radiological response were secondary endpoints. Median PFS was reported as 238 days (95% CI 221-281) in the TACE plus sorafenib arm and 235 days (95% CI 209-322) in the TACE plus placebo arm, without any statistically significant hazard ratio (HR) when comparing between groups 0.99 (95% CI 0.77-1.27; p = 0.94). Median OS was reported as 631 days (95% CI 473-879) in the TACE plus sorafenib arm and 598 days (95% CI 500-697) in the TACE plus placebo arm, again without any statistically significant difference between groups (HR 0.91, 95% CI 0.67-1.24; p = 0.57).

The co-primary endpoints for TACTICS included TACE-specific PFS and OS. Progression was defined as unTACE-able progression, which is the inability of a patient to further receive or benefit from TACE for reasons that included intrahepatic tumour progression (defined as 25% increase of viable area in the sum of the five largest intrahepatic lesions compared to baseline), transient deterioration of liver function to Child-Pugh grade C immediately after TACE, the appearance of macrovascular invasion or new extrahepatic metastases. Tumour response was evaluated according to RECICL (Response Evaluation Criteria in Cancer of the Liver).[17] Median PFS was reported as significantly longer in the TACE plus sorafenib group than in the TACE alone group (25.2 *vs.* 13.5 months; HR = 0.59; 95% CI 0.41–0.87; p = 0.006). However, median OS in the TACE plus sorafenib group was 36.2 months (95% CI 30.5–44.1), whereas in the TACE-alone group it was 30.8 months (95% CI 23.5–40.8), with no statistically significant difference between the two groups (HR = 0.861; 95% CI 0.607-1.223; p = 0.40).

TACE-2 and TACTICS complied with the Declaration of Helsinki and applicable local regulations. Ethics committees at all participating institutions approved the protocol and all patients provided written informed consent.

Statistical analysis

For the purposes of this study, only patients with radiological progression were included. These patients were categorised into three groups: target or non-target lesion progression (TNTLP), new intrahepatic lesions (NIH), and new extrahepatic lesions (NEH). Only one type of pattern of progression was recorded for each patient. Note that TNTLP group refers to patients who had progression of intrahepatic lesions that were present at randomisation in the TACE-2 and TACTICS trials. Extrahepatic disease was an exclusion criterion for both trials and therefore patients only qualified for progression when new extrahepatic lesions were identified and qualified as NEH. The timing of first disease progression was categorised as <6 months, 6–12 months, or >12 months.

The HAP score was derived from the case report forms. It is a composite prognostic scoring system based on alpha-fetoprotein (AFP), bilirubin, albumin, and tumour diameter, and validated in patients treated with TACE.[9]

Baseline characteristics were summarised according to pattern of progression using frequencies and percentages for categorical variables, and medians with ranges for continuous variables. Comparisons between progression pattern groups were performed using chi-square tests for categorical variables and Kruskal-Wallis tests for continuous variables.

The chi-square test was used to assess associations between adverse HAP scores and pattern of progression. It was also applied to evaluate whether there was an association between treatment and pattern of progression.

PPOS was defined as the time from first documented progression to death. Patients who were alive at the last follow-up or lost to follow-up were censored at their last known alive date. PPOS was evaluated using Kaplan-Meier methods, with a survival curve figure displaying the differences in survival across the three progression pattern groups. Summary statistics, including median PPOS and 12-month PPOS estimates, are reported.

A Cox proportional hazards regression model was fitted to assess the independent effect of progression pattern and HAP score on PPOS. A multivariable model was performed to examine the effect of progression on survival after adjusting for HAP score. Hazard ratios (HR) with 95% CIs are reported.

Given the exploratory nature of this analysis, no adjustments for multiple testing were applied to pairwise comparisons between progression pattern groups. Statistical significance was set at $\alpha = 0.05$ for all tests. All analyses were performed using STATA 18.5.[18]

Results

TACE-2 recruited 313 patients from 20 UK sites and TACTICS recruited 156 patients from 33 sites in Japan. In total, 285 patients had radiological progression at the final analysis and were included in this study. There were no clear differences in baseline characteristics comparing those who progressed in the two treatment arms (Table S1). Among the 285 progressors, 167 (59%) progressed with NIH, 86 (30%) with TNTLP and 32 (11%) developed NEH metastases (Table 1). When comparing the pattern of progression groups, there were no statistically significant differences in sex, age, disease focality, or Child–Pugh grade.

Patients in the NEH group tended to have a larger median target 1 lesion (6.2 cm) compared with 3.9 cm and 3.5 cm in the TNTLP and NIH groups, respectively. Similarly, mean AFP was higher in the NEH group (5,507 ng/ml) than in the TNTLP (1,580 ng/ml) and NIH (1,837 ng/ml) groups, although this difference was not statistically significant. Adverse HAP scores were also overrepresented in the NEH group with 34% having a HAP score of C or D compared with 19% and 22% for the TNTLP and NIH groups, respectively; however, this difference was not statistically significant. Since liver function seemed similar across the three groups, the difference in HAP score is likely attributable to the size of the largest tumour and AFP level. There were also statistical differences in bilirubin, ECOG performance status and time from randomisation to progression, but this was most likely due to the small numbers of patients in the NEH group skewing the distribution of the data and thereby conferring a significant p value.

Patients with NEH progression had shorter median PPOS compared to TNTLP and NIH groups, 7.0 months compared to

Table 1. Baseline characteristics by pattern of disease progression.

Baseline characteristics	Target or non-target lesion<br>n = 86	New lesion in the liver<br>n = 167	New lesion outside the liver<br>n = 32	*p* value
Sex				
Male	67 (78%)	133 (80%)	29 (91%)	
Female	19 (22%)	34 (20%)	3 (9%)	0.284
Age (years)				
Median (range)	71 (46 to 86)	70 (36 to 86)	67 (50 to 81)	0.0966
Bilirubin (µmol/L)				
Median (range)	14 (4.0 to 50)	14 (3 to 39.0)	9.5 (4.0 to 29.0)	0.0108
Albumin (µmol/L)				
Median (range)	40.5 (29.0 to 48.0)	39.0 (29.0 to 57.0)	38.0 (26.0 to 47.0)	0.1089
RECIST: Target lesion 1 (cm)				
Median (range)	3.9 (1.0 to 23.0)	3.5 (1.2 to 14.6)	6.1 (1.2 to 18.1)	0.0006
RECIST: Target lesion 2 (cm)				
Median (range)	2 (0.5 to 7.5)	1.8 (0.5 to 10.8)	2.4 (0.6 to 5.0)	0.1470
AFP (kU/L)				
Median (range)	14.8 (0.8 to 71,000)	15.8 (1.0 to 100,199.0)	18.4 (1 to 100,000.0)	0.5971
Time from randomisation to progression (months)				
Median (range)	4.7 (1.0 to 32.8)	7.6 (0.8 to 69.7)	5.1 (1.7 to 59.7)	0.0002
ECOG performance status				
0	59 (69%)	134 (80%)	19 (59%)	
1	27 (31%)	33 (20%)	12 (38%)	
Not known	0 (0%)	0 (0%)	1 (3%)	0.025
Disease focality nodules				
1	20 (23%)	39 (23%)	12 (38%)	
2	23 (27%)	42 (25%)	6 (19%)	
3	14 (16%)	32 (19%)	5 (16%)	
>3	26 (30%)	53 (32%)	9 (28%)	
Not known	3 (3%)	1 (1%)	0 (0%)	0.769
HAP score				
HAP A	32 (37%)	66 (40%)	11 (34%)	
HAP B	37 (43%)	63 (38%)	10 (31%)	
HAP C	12 (14%)	32 (19%)	9 (28%)	
HAP D	4 (5%)	4 (2%)	2 (6%)	
Not available	1 (1%)	2 (1%)	0 (0%)	0.507
Child-Pugh				
A	86 (100%)	163 (98%)	31 (97%)	
B	0 (0%)	4 (2%)	1 (3%)	0.319
ALBI grade				
1	46 (53%)	77 (46%)	16 (50%)	
2	40 (47%)	89 (53%)	16 (50%)	
Not known	0 (0%)	1 (1%)	0 (0%)	0.560

AFP, alpha-fetoprotein; ALBI, albumin-bilirubin; HAP, hepatoma arterial embolisation prognostic.

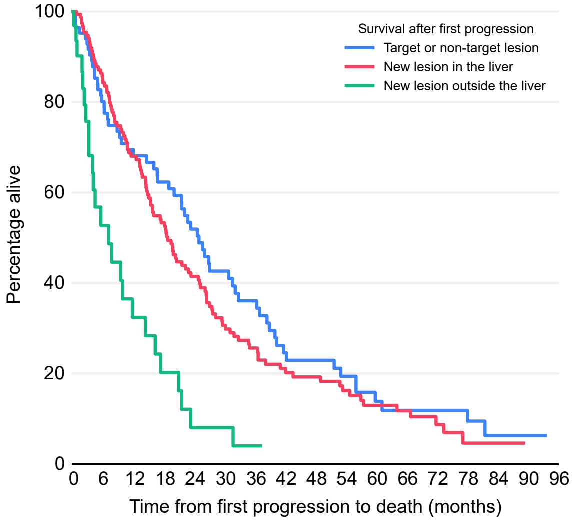

N° at risk																	
Target or non-target lesion	86	62	49	43	34	26	22	14	13	11	7	5	5	4	2	1	0
New lesion in the liver	167	118	88	68	50	36	30	22	20	15	11	10	5	1	1	0	0
New lesion outside the liver	32	13	8	5	2	2	1	0	0	0	0	0	0	0	0	0	0

Fig. 1. Kaplan-Meier survival curves of post progression overall survival stratified by pattern of progression.

24.8 months and 18.7 months, respectively (Fig. 1). This is reflected in the hazard ratio from the univariate analysis, which showed a significantly increased risk of death for this subgroup compared with the TNTLP group, at 3.11 (95% CI 1.93–5.01; p <0.001), the latter having the lowest risk of death (Table 2). This poorer prognosis for the NEH group was independent of the HAP score, as demonstrated by the multivariate analysis, which adjusted for HAP score, with the NEH group having an increased risk of death at 3.05 (95% CI 1.88-4.94; p <0.001) (Table 2). We additionally performed a further multivariate analysis of the pattern of progression group with other known prognostic variables including Child-Pugh score, ALBI (albumin-bilirubin) grade, AFP, largest tumour size and disease focality (Table 3), demonstrating that the poorer prognosis for the NEH group remains independent from these other factors.

Time from randomisation to progression did not affect post progression survival. When grouped into three groups by time from randomisation to progression (less than 6 months, between 6-to-12 months, and more than 12 months), there was no statistically significant difference in PPOS (Fig. S1, Table S2). The 6–12-month and >12-month patient groups showed no statistically significant difference in HRs compared with the <6-month group, with HRs of 0.87 (95% CI 0.62–1.21) and 0.86 (95% CI 0.59–1.26), respectively (Table S3). There was no evidence that sorafenib influenced the pattern of progression, with a similar proportion progressing in each category (Table 4). Additionally, the effect of treatment on PPOS did not differ significantly across progression pattern groups (Fig. 2, Table 4). For all three pattern of progression groups, HRs comparing TACE plus placebo with TACE plus sorafenib were not statistically significant: TNTLP group HR 0.84 (95% CI 0.51–1.39; p = 0.50), NIH group HR 0.88 (95% CI 0.60–1.28; p = 0.49), and NEH group HR 1.15 (95% CI 0.52–2.55; p = 0.74).

Discussion

In this large, multicentre, prospective study, we have demonstrated that pattern of progression with NEH is associated with worse PPOS than NIH or growth of pre-existing lesions in a TACE-treated population of patients. Furthermore, we have shown that addition of sorafenib to TACE treatment is not associated with improved PPOS within these three patterns of progression.

The pattern of progression has been shown to be of prognostic importance in the context of a variety of treatment modalities for HCC. Reig *et al.* demonstrated that in 43 patients who had progressive disease after treatment with sorafenib, those who progressed with NEH had worse post-progression survival compared to their counterparts who progressed with NIH or growth of pre-treatment intrahepatic lesions.[19] Pattern of progression has also been shown to be an independent prognostic factor after treatment with other systemic anticancer therapies including sorafenib, atezolizumab-bevacizumab, regorafenib, tivantinib and ramucirumab,[20–27] as well as in selective internal radioembolisation therapy.[28] It has therefore been suggested that pattern of progression should be considered as a stratification factor for subsequent clinical trials.

However, there are few studies that have explored pattern of progression in the context of embolic therapy. Song *et al.* conducted a single-centre retrospective cohort study examining TACE in HCC not amenable to ablation.[11] They identified 84 patients who progressed post-TACE, of whom only two developed NEH lesions; therefore, the prognostic relevance of NEH could not be reliably assessed due to the small number of events. Labeur *et al.* reported a single retrospective study

Table 2. Post-progression overall survival by pattern of progression and HAP score (multivariable *vs.* univariable Cox regression).

Post-progression overall survival	Univariable Cox model		Multivariable Cox model*	
	HR (95% CI)	*p* value	HR (95% CI)	*p* value
Pattern of progression				
Target or non-target lesion	1.00 (ref)		1.00 (ref)	
New lesion in the liver	1.17 (0.86 to 1.60)	0.31	1.20 (0.88 to 1.65)	0.25
New lesion outside the liver	3.11 (1.93 to 5.01)	p <0.001	3.04 (1.88 to 4.93)	p <0.001
HAP score				
HAP A	1.00 (ref)		1.00 (ref)	
HAP B	1.30 (0.95 to 1.80)	0.104	1.28 (0.93 to 1.77)	0.13
HAP C	2.09 (1.44 to 3.05)	p <0.001	1.97 (1.35 to 2.88)	p <0.001
HAP D	13.89 (5.69 to 33.87)	p <0.001	15.27 (6.22 to 37.50)	p <0.001

HAP, hepatoma arterial embolisation prognostic; HR, hazard ratio.

Table 3. Post-progression overall survival by pattern of progression, Child-Pugh score, ALBI grade, AFP, largest tumour size, disease focality (multivariable *vs.* univariable Cox regression).

Patterns of progression	Univariable Cox model		Multivariable Cox model*	
	HR (95% CI)	*p* value	HR (95% CI)	*p* value
Target or non-target lesion	1.00 (ref)		1.00 (ref)	
New lesion in the liver	1.17 (0.86 to 1.60)	0.31	1.25 (0.90 to 1.72)	0.182
New lesion outside the liver	3.11 (1.93 to 5.01)	*p* <0.001	2.47 (1.49 to 4.09)	*p* <0.001
Child-Pugh score				
A	1.00 (ref)		1.00 (ref)	
B	1.16 (0.43 to 3.12)	0.774	1.03 (0.37 to 2.83)	0.959
ALBI grade				
1	1.00 (ref)		1.00 (ref)	
2	1.51 (1.15 to 2.00)	0.003	1.56 (1.17 to 2.08)	0.003
AFP	1.00 (1.00 to 1.00)	0.308	1.00 (1.00 to 1.00)	0.689
Largest tumour	1.10 (1.06 to 1.14)	*p* <0.001	1.08 (1.04 to 1.13)	*p* <0.001
Disease focality				
1	1.00 (ref)		1.00 (ref)	
2	1.16 (0.77 to 1.76)	0.466	1.34 (0.88 to 2.04)	0.171
3	0.95 (0.62 to 1.45)	0.804	1.02 (0.66 to 1.57)	0.941
>3	1.03 (0.71 to 1.51)	0.873	1.27 (0.85 to 1.89)	0.238

AFP, alpha-fetoprotein; ALBI, albumin-bilirubin; HR, hazard ratio.

including 105 patients with radiological progression post-TACE and demonstrated that the 26 patients with NEH progression had worse median post-progression survival (4.7 months, 95% CI 3.4–6.0) compared to patients with intrahepatic progression (10.3 months, 95% CI 7.8–12.9).[12]

Our combined analysis of the TACE-2 and TACTICS trials is the first prospective multicentre study to demonstrate that different patterns of progression are associated with different PPOS after TACE treatment. This adds to the existing literature, indicating that pattern of progression is an independent post-progression prognostic factor across a wide range of treatments for HCC.[20–28] Both TACE-2 and TACTICS failed to meet their primary endpoints, and neither demonstrated an overall survival benefit with the addition of sorafenib to TACE compared with TACE alone. Similarly, the SPACE trial[29] also failed to show a benefit for sorafenib, as did other phase III trials evaluating alternative tyrosine kinase inhibitors including orantinib and brivanib.[30,31] More recently immune checkpoint inhibitors have been evaluated in combination with TACE in EMERALD-1 and LEAP-12 studies.[32,33] Both studies demonstrated an improvement in progression-free survival with the addition of durvalumab and bevacizumab or pembrolizumab and lenvatinib. The final overall survival analyses require further follow-up. Neither study has reported pattern of progression or post-progression survival but, given the distinct microenvironment of the liver and metastatic niche, it will be of interest to explore this in due course.

This study has several limitations. First, information on post-progression therapy was not collected for both studies, and the application of further therapy may influence post-progression survival. For example, patients with intrahepatic progression may have been eligible to receive further locoregional therapy whereas this would not be the case for those with extrahepatic progression. However, this does not detract from the fundamental finding that extrahepatic progression is associated with worse overall survival. Second, investigators only reported one type of progression, and it is possible that some patients had a mixed pattern of progression that was not captured on the case report form. Our assumption is that in those cases where there was a mixed pattern of progression, extrahepatic disease would have been documented in favour of intrahepatic progression and that new hepatic disease would be reported in favour of target lesion progression. Finally, we have only evaluated the prognostic relevance of radiological pattern of progression and not the impact of TACE refractoriness. TACE refractoriness due to decompensated liver disease or lack of response has already been confirmed as an adverse factor in multiple studies.

Table 4. Multivariable Cox regression analysis of post-progression overall survival by progression pattern and treatment.

Post-progression overall survival	n/N deaths (%)	Median PPOS (in months)	12 months PPOS	Multivariable Cox model*	
				HR (95% CI)	*p* value
Target or non-target lesion					
TACE + control	33/45 (73%)	21.4	64%	1.00 (ref)	
TACE + sorafenib	29/41 (71%)	26.7	73%	0.84 (0.50 to 1.39)	0.49
New lesion in the liver					
TACE + control	61/83 (73%)	15.9	61%	1.00 (ref)	
TACE + sorafenib	55/84(65%)	21.5	75%	0.88 (0.60 to 1.28)	0.50
New lesion outside the liver					
TACE + control	13/17 (76%)	7.6	37%	1.00 (ref)	
TACE + sorafenib	12/15 (80%)	7	27%	1.15 (0.52 to 2.55)	0.74

HAP, hepatoma arterial embolisation prognostic; HR, hazard ratio; PPOS, post-progression overall survival; TACE, transarterial chemoembolisation.
*Adjusted for HAP score.

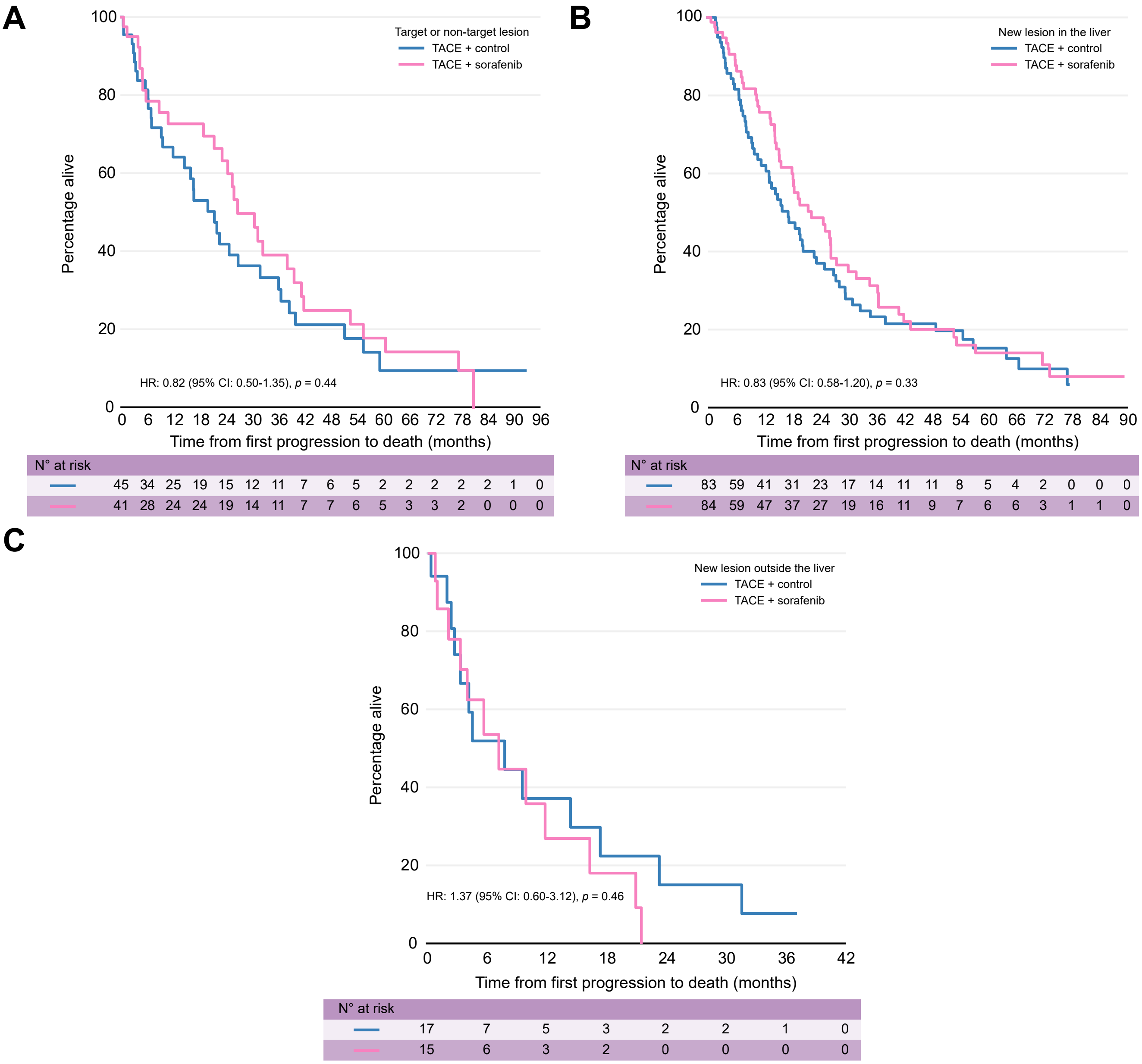

Fig. 2. Kaplan Meier survival curves of post-progression overall survival by treatment stratified by progression type. (A) target or non-target lesion, (B) new lesion in the liver and (C) new lesion outside the liver.

In summary, we have shown for the first time in a large prospective multicentre study, that there appears to be an association between patterns of progression and post-progression survival in a TACE-treated population, with patients progressing with extrahepatic disease being associated with a worse prognosis post-progression. Given around 50% patients receive locoregional therapy prior to entry into trials of systemic therapy,[34,35] our study underlines the importance of including extrahepatic spread as a key stratification factor in clinical trials of systemic therapy, which has been reflected in the design of trials in HCC, such as the IMBRAVE150 trial.

Affiliations

[1]*Department of Medical Oncology, Royal Free Hospital, London, UK;* [2]*Cancer Research UK & UCL Cancer Trials Centre, University College London, 90 Tottenham Court Road, London W1T 4TJ, UK;* [3]*Department of Gastroenterology and Hepatology, Kindai University Faculty of Medicine 377-2, Ohno-Higashi, Osaka-Sayama Osaka, Japan;* [4]*University of Birmingham and University Hospitals Birmingham NHS Foundation Trust, Birmingham, UK;* [5]*Department of Oncology, King's College Hospital, London, UK;* [6]*University of Liverpool and The Clatterbridge Cancer Centre NHS Foundation Trust, Liverpool, UK;* [7]*UCL Cancer Institute, University College London, UK*

Abbreviations

AFP, alpha-fetoprotein; cTACE, conventional transarterial chemoembolisation; DEB-TACE, drug-eluting bead transarterial chemoembolisation; HAP, hepatoma arterial embolisation prognostic; HCC, hepatocellular carcinoma; HR, hazard ratios; IHG, intrahepatic lesion growth; NEH, new extrahepatic lesions; NIH, new intrahepatic lesions; OS, overall survival; PFS, progression-free survival; PPOS, post-progression overall survival; TACE, transarterial chemoembolisation.

Financial support

TM is funded by National Institute for Health Research. The TACE 2 trial was sponsored by UCL and funded by MSD and Biocompatibles. The TACTICS trial was funded by Bayer Yakuhin, Ltd.

Conflicts of interest

J. S. J. Yuan-Doré reports no conflicts of interest. M. Rashid reports no conflicts of interest. K. Ueshima reports receiving lecture fees from Eisai, ONO, BMS, AstraZeneca and Chugai; research funding from Chugai. A. Lopes reports no conflicts of interest. Y. T. Ma reports grants from Eisai, AstraZeneca/Merck, Faron Pharmaceuticals, Mina Therapeutics; consulting fees from Roche, AstraZeneca/MedImmune, Faron Pharmaceuticals, Incyte. P. J. Ross reports grants from Sonofi; consulting fees from Amgen, Takeda, BMS, Taiho Oncology; lecture fees from AstraZeneca, Bayer, Eisai, Amgen,Takeda, Taiho Oncology, BMS, Merck, Merck Serono; support for travel from Takeda, Merck Serono; stock from Perci Health. D. Palmer reports grants from BMS, Sirtex, Nucana, Medannex; consulting fees from MSD, BMS, AstraZeneca, Sirtex, Taiho, Jazz, Viatris, Nucana, Medannex, Servier, Pfizer. M. Kudo reports Consultancy from MSD, BMS, Chugai, Eisai, Roche and Ono pharmaceutical; lecture fees from Eisai, BMS, and Chugai; Research grant from Chugai, Otsuka, Takeda, Taiho, MSD, Eisai, AbbVie, BMS. T. Meyer reports Consultancy: Roche, Astra Zeneca, Signant Health, GreyWolf, Guerbet, Geneos, Eisai, Beigene, MSD. Research Funding: MSD, Bayer, Boston Scientific.
Please refer to the accompanying ICMJE disclosure forms for further details.

Authors' contributions

Concept and design: JSJYD, MR, MK, TM. Collection of data: KU, YTM, PR, DP, MK, TM. Drafting of manuscript: JSY, MR, TM. Approval of final manuscript: All.

Data availability

The data that support the findings of this study are available from the corresponding author upon reasonable request.

Supplementary data

Supplementary data to this article can be found online at https://doi.org/10.1016/j.jhepr.2026.101791.

References

Author names in bold designate shared co-first authorship

[1] European Association for the Study of the Liver. EASL Clinical Practice Guidelines on the management of hepatocellular carcinoma. J Hepatol 2025;82:315–374. https://doi.org/10.1016/j.jhep.2024.08.028.
[2] Suddle A, Reeves H, Hubner R, et al. British Society of Gastroenterology guidelines for the management of hepatocellular carcinoma in adults. Gut 2024;73:1235–1268. https://doi.org/10.1136/gutjnl-2023-331695.
[3] Reig M, Forner A, Rimola J, et al. BCLC strategy for prognosis prediction and treatment recommendation: the 2022 update. J Hepatol 2022;76:681–693. https://doi.org/10.1016/j.jhep.2021.11.018.
[4] Vogel A, Chan SL, Dawson LA, et al. Hepatocellular carcinoma: ESMO Clinical Practice Guideline for diagnosis, treatment and follow-up. Ann Oncol 2025;36:491–506. https://doi.org/10.1016/j.annonc.2025.02.006.
[5] Llovet JM, Bruix J. Systematic review of randomized trials for unresectable hepatocellular carcinoma: chemoembolization improves survival. Hepatology 2003;37:429–442. https://doi.org/10.1053/jhep.2003.50047.
[6] Tsochatzis EA, Meyer T, O'Beirne J, et al. Transarterial therapies for hepatocellular carcinoma (HCC): a long way towards standardization. J Hepatol 2013;58:194. https://doi.org/10.1016/j.jhep.2012.08.028.
[7] Golfieri R, Giampalma E, Renzulli M, et al. Randomised controlled trial of doxorubicin-eluting beads vs conventional chemoembolisation for hepatocellular carcinoma. Br J Cancer 2014;111:255–264. https://doi.org/10.1038/bjc.2014.199.
[8] Meyer T, Kirkwood A, Roughton M, et al. A randomised phase II/III trial of 3-weekly cisplatin-based sequential transarterial chemoembolisation vs embolisation alone for hepatocellular carcinoma. Br J Cancer 2013;108:1252–1259. https://doi.org/10.1038/bjc.2013.85.
[9] Kadalayil L, Benini R, Pallan L, et al. A simple prognostic scoring system for patients receiving transarterial embolisation for hepatocellular cancer. Ann Oncol 2013;24:2565. https://doi.org/10.1093/annonc/mdt247.
[10] Han G, Berhane S, Toyoda H, et al. Prediction of survival among patients receiving transarterial chemoembolization for hepatocellular carcinoma: a response-based approach. Hepatology 2020;72:198. https://doi.org/10.1002/hep.31022.
[11] Song YG, Shin SW, Cho SK, et al. Transarterial chemoembolization as first-line therapy for hepatocellular carcinomas infeasible for ultrasound-guided radiofrequency ablation: a retrospective cohort study of 116 patients. Acta Radiol 2015;56:70–77. https://doi.org/10.1177/0284185114520857.
[12] Labeur TA, Takkenberg RB. Klümpen H.-J.et al. Reason of discontinuation after transarterial chemoembolization influences survival in patients with hepatocellular carcinoma. Cardiovasc Intervent Radiol 2019;42:230–238. https://doi.org/10.1007/s00270-018-2118-6.
[13] Meyer T, Fox R, Ma YT, et al. Sorafenib in combination with transarterial chemoembolisation in patients with unresectable hepatocellular carcinoma (TACE 2): a randomised placebo-controlled, double-blind, phase 3 trial. Lancet Gastroenterol Hepatol 2017;2:565–575. https://doi.org/10.1016/S2468-1253(17)30156-5.
[14] Kudo M, Ueshima K, Ikeda M, et al. Final results of TACTICS: a randomized, prospective trial comparing transarterial chemoembolization plus sorafenib to transarterial chemoembolization alone in patients with unresectable hepatocellular carcinoma. Liver Cancer 2022;11:354–367. https://doi.org/10.1159/000522547.
[15] **Kudo M**, **Ueshima K**, Ikeda M, et al. Randomised, multicentre prospective trial of transarterial chemoembolisation (TACE) plus sorafenib as compared with TACE alone in patients with hepatocellular carcinoma: TACTICS trial. Gut 2020;69:1492–1501. https://doi.org/10.1136/gutjnl-2019-318934.
[16] Eisenhauer EA, Therasse P, Bogaerts J, et al. New response evaluation criteria in solid tumours: revised RECIST guideline (version 1.1). Eur J Cancer 2009;45:228–247. https://doi.org/10.1016/j.ejca.2008.10.026.
[17] Kudo M, Ikeda M, Ueshima K, et al. Response evaluation criteria in cancer of the liver version 6 (response evaluation criteria in cancer of the liver 2021 revised version). Hepatol Res 2022;52:329–336. https://doi.org/10.1111/hepr.13746.
[18] StataCorp. Stata Stat Softw Release 2023;18.
[19] Reig M, Rimola J, Torres F, et al. Postprogression survival of patients with advanced hepatocellular carcinoma: rationale for second-line trial design. Hepatology 2013;58:2023–2031. https://doi.org/10.1002/hep.26586.
[20] Talbot T, D'Alessio A, Pinter M, et al. Progression patterns and therapeutic sequencing following immune checkpoint inhibition for hepatocellular carcinoma: an international observational study. Liver Int 2023;43:695–707. https://doi.org/10.1111/liv.15502.
[21] Campani C, Vallot A, Ghannouchi H, et al. Impact of radiological response and pattern of progression in patients with HCC treated by atezolizumab-bevacizumab. Hepatology 2024;79:49. https://doi.org/10.1097/HEP.0000000000000636.
[22] Bruix J, Qin S, Merle P, et al. Regorafenib for patients with hepatocellular carcinoma who progressed on sorafenib treatment (RESORCE): a randomised, double-blind, placebo-controlled, phase 3 trial. The Lancet 2017;389:56–66. https://doi.org/10.1016/S0140-6736(16)32453-9.
[23] Rimassa L, Assenat E, Peck-Radosavljevic M, et al. Tivantinib for second-line treatment of MET-high, advanced hepatocellular carcinoma (METIV-HCC): a final analysis of a phase 3, randomised, placebo-controlled study. Lancet Oncol 2018;19:682–693. https://doi.org/10.1016/S1470-2045(18)30146-3.
[24] Reig M, Galle PR, Kudo M, et al. Pattern of progression in advanced hepatocellular carcinoma treated with ramucirumab. Liver Int 2021;41:598–607. https://doi.org/10.1111/liv.14731.
[25] de la Torre-Aláez M, Reig M. The 'BCLC upon progression' classification: after repeated validation, it is time to incorporate it in trial design. Liver Int 2024;44:1273–1275. https://doi.org/10.1111/liv.15850.
[26] Iavarone M, Cabibbo G, Biolato M, et al. Predictors of survival in patients with advanced hepatocellular carcinoma who permanently discontinued sorafenib. Hepatology 2015;62:784. https://doi.org/10.1002/hep.27729.
[27] Ogasawara S, Chiba T, Ooka Y, et al. Post-progression survival in patients with advanced hepatocellular carcinoma resistant to sorafenib. Invest New Drugs 2016;34:255–260. https://doi.org/10.1007/s10637-016-0323-1.

[28] de la Torre-Aláez M, Jordán-Iborra C, Casadei-Gardini A, et al. The pattern of progression defines post-progression survival in patients with hepatocellular carcinoma treated with SIRT. Cardiovasc Intervent Radiol 2020;43:1165–1172. https://doi.org/10.1007/s00270-020-02444-2.

[29] **Lencioni R**, **Llovet JM**, Han G, et al. Sorafenib or placebo plus TACE with doxorubicin-eluting beads for intermediate stage HCC: the SPACE trial. J Hepatol 2016;64:1090–1098. https://doi.org/10.1016/j.jhep.2016.01.012.

[30] Kudo M, Cheng A-L, Park J-W, et al. Orantinib versus placebo combined with transcatheter arterial chemoembolisation in patients with unresectable hepatocellular carcinoma (ORIENTAL): a randomised, double-blind, placebo-controlled, multicentre, phase 3 study. Lancet Gastroenterol Hepatol 2018;3:37–46. https://doi.org/10.1016/S2468-1253(17)30290-X.

[31] Kudo M, Han G, Finn RS, et al. Brivanib as adjuvant therapy to transarterial chemoembolization in patients with hepatocellular carcinoma: a randomized phase III trial. Hepatology 2014;60:1697–1707. https://doi.org/10.1002/hep.27290.

[32] Kudo M, Ren Z, Guo Y, et al. Transarterial chemoembolisation combined with lenvatinib plus pembrolizumab versus dual placebo for unresectable, non-metastatic hepatocellular carcinoma (LEAP-012): a multicentre, randomised, double-blind, phase 3 study. The Lancet 2025;405:203–215. https://doi.org/10.1016/S0140-6736(24)02575-3.

[33] Sangro B, Kudo M, Erinjeri JP, et al. Durvalumab with or without bevacizumab with transarterial chemoembolisation in hepatocellular carcinoma (EMERALD-1): a multiregional, randomised, double-blind, placebo-controlled, phase 3 study. The Lancet 2025;405:216–232. https://doi.org/10.1016/S0140-6736(24)02551-0.

[34] Llovet JM, Kudo M, Merle P, et al. Lenvatinib plus pembrolizumab versus lenvatinib plus placebo for advanced hepatocellular carcinoma (LEAP-002): a randomised, double-blind, phase 3 trial. Lancet Oncol 2023;24:1399–1410. https://doi.org/10.1016/S1470-2045(23)00469-2.

[35] Finn RS, Qin S, Ikeda M, et al. Atezolizumab plus bevacizumab in unresectable hepatocellular carcinoma. N Engl J Med 2020;382:1894–1905. https://doi.org/10.1056/NEJMoa1915745.

Keywords: hepatocellular carcinoma; transarterial chemoembolization; trial design; stratification factor.

Received 17 September 2025; received in revised form 5 February 2026; accepted 12 February 2026; Available online 25 February 2026

Journal of Hepatology, Volume *8*

Supplemental information

Pattern of progression and post-progression survival following trans-arterial embolisation: An analysis of the TACE-2 and TACTICS trials

Jack Shi Jie Yuan-Doré, Memuna Rashid, Kazuomi Ueshima, Andre Lopes, Yuk Ting Ma, Paul Ross, Daniel Palmer, Masatoshi Kudo, and Tim Meyer

Pattern of progression and post-progression survival following transarterial embolisation: An analysis of the TACE-2 and TACTICS trials

Jack Shi Jie Yuan, Memuna Rashid, Kazuomi Ueshima, Andre Lopes, Yuk Ting Ma, Paul J Ross, Daniel Palmer, Masatoshi Kudo, Tim Meyer

Table of contents

Table S1……………………………………………………………………………………2

Table S2……………………………………………………………………………………3

Table S3……………………………………………………………………………………4

Table S4……………………………………………………………………………………4

Fig. S1…………………………………………………………………………………......5

Table S1: Baseline characteristics among patients with disease progression by treatment group

Baseline characteristics	TACE + Sorafenib N=140	TACE Control N=145
**Sex**		
Male	112 (80%)	117 (81%)
Female	28 (20%)	28 (19%)
**Age (years)**		
Median(range)	70.0 (36.0 to 85.0)	71.0 (46.0 to 86.0)
**Bilirubin (μmol/l)**		
Median(range)	13.0 (3.0 to 36.0)	14.0 (4 to 50)
**Albumin (g/l)**		
Median(range)	40.0 (29.0 to 50.0)	39.0 (26.0 to 57.0)
**RECIST: Target Lesion 1 (cm)**		
Median (range)	4.0 (1.0 to 19.6)	3.5 (1.0 to 23.0)
**RECIST: Target Lesion 2 (cm)**		
Median (range)	1.9 (0 .8 to 7.5)	2.0 (0.5 to 10.8)
**AFP (kU/L)**		
Median (mean) (range)	15.9 (2364.0) (0.8 to 100199.0)	14.8 (1988.6) (1.3 to 100000.0)
**Time from randomisation to progression(months)**		
Median (range)	7.1 (1.0 to 69.7)	5.4 (0.8 to 46.0)
**ECOG PS**		
0	104 (74%)	108 (74%)
1	35 (25%)	37 (26%)
Not known	1 (1%)	0 (0%)
**Disease Focality nodules**		
1	34 (24%)	37 (26%)
2	36 (26%)	35 (24%)
3	28 (20%)	23 (16%)
>3	41 (29%)	47(32%)
Not known	1(1%)	3(2%)
**HAP score**		
HAP A	59 (42%)	50 (34%)
HAP B	49 (35%)	61 (42%)
HAP C	26 (19%)	27 (19%)
HAP D	6 (4%)	4 (3%)

Not available	0 (0%)	3 (2%)

Table S2. Descriptive analysis of post-progression overall survival stratified by time from randomisation to progression

Time from randomisation to progression	N	N deaths (%)	Median PPOS (in months)	12 months PPOS
<6 months	144	118 (82%)	17.3	62%
≥6 months to ≤12 months	78	50 (64%)	23.2	72%
>12 months	63	35 (56%)	19.3	59%

*PPOS: Post-progression overall survival

Table S3. Post-progression overall survival by time from randomisation to progression groups (Univariable vs. Multivariable Cox Regression)

**Time from randomisation to progression**	**Univariable Cox Model**		**Multivariable Cox Model***	
	**HR (95% CI)**	**P-value**	**HR (95% CI)**	**P-value**
<6 months	1.00 (ref)		1.00 (ref)	
≥6 months to ≤12 months	0.87 (0.62 to 1.21)	0.41	0.87 (0.62 to 1.21)	0.41
>12 months	0.86 (0.59 to 1.26)	0.45	0.95 (0.65 to 1.3940)	0.80
HAP score				
HAP A	1.00 (ref)		1.00 (ref)	
HAP B	1.3029 (0.95 to 1.80)	0.104	1.30 (0.94 to 1.79)	0.11
HAP C	2.09 (1.44 to 3.05)	P < 0.001	2.08 (1.42 to 3.03)	p < 0.001
HAP D	13.89 (5.69 to 33.87)	P < 0.001	14.00 (5.71 to 34.33)	p < 0.001

* Adjusted for HAP score

Table S4. Comparison of the multivariable analysis of the TACE-2 and TACTICS trial

**Post-Progression Overall Survival (Multivariable model)**	TACTICS		TACE-2	
	HR (95% CI	P-value	HR (95% CI)	P-value
**Pattern of progression**				
Target or non-target lesion	1.00 (ref)		1.00 (ref)	
New lesion in the liver	1.45 (0.97 to 2.17	0.072	0.86 (0.51 to 1.43)	0.56
New lesion outside the liver	9.29(3.14 to 27.52)	p <0.001	1.35 (0.74 to 2.45)	0.33
HAP score				
HAP A	1.00 (ref)		1.00 (ref)	
HAP B	1.25 (0.84 to 1.85)	0.276	1.40 (0.79 to 2.46)	0.25
HAP C	1.48 (0.84 to 2.60)	0.176	2.55(1.41 to 4.60)	0.002
HAP D	-	-	9.04 (3.42 to 23.88)	p < 0.001

Fig. S1 – Kaplan Meier plot for post-progression overall survival stratified by time from randomisation to progression

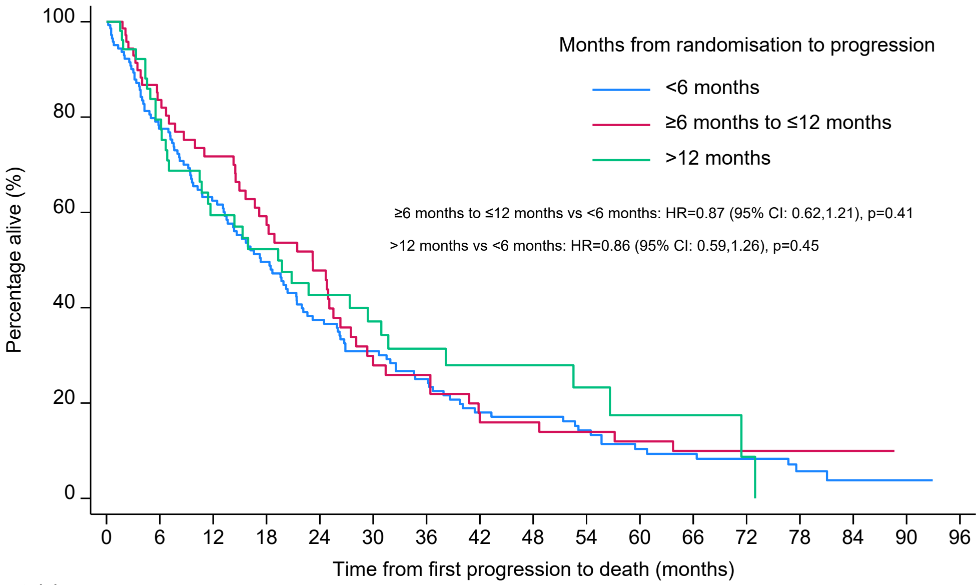

Number at risk																	
<6 months	144	104	80	62	46	37	30	20	18	15	10	9	7	4	2	1	0
≥6 months to ≤12 months	78	52	40	32	24	14	13	8	8	7	6	4	2	1	1	0	0
>12 months	63	37	25	22	16	13	10	8	7	4	2	2	1	0	0	0	0
